# Preventing EBV immune evasion

**Published:** 2014-04

Latent infection with Epstein-Barr virus (EBV) is associated with several human cancers, including nasopharyngeal cancer. During latent EBV infections, the glycine-alanine repeat (GAr) domain of the EBV nuclear antigen-1 (EBNA1) inhibits translation of *EBNA1* mRNA, thereby suppressing the production of antigenic peptides that the human immune system recognises as foreign. Here, Blondel et al. establish a yeast-based assay that recapitulates GAr-mediated suppression of *EBNA1* mRNA translation and use it to identify doxorubicin and its active analogues as compounds that specifically interfere with GAr-mediated suppression of translation. Importantly, in mammalian cells, doxorubicin and its analogues stimulate EBNA1 expression in a GAr-dependent manner and overcome the GAr-dependent restriction of antigen presentation. Together, these results validate the yeast-based assay as an effective cell-based screening approach for compounds that interfere with EBV immune evasion and that might, therefore, provide treatments for EBV-related diseases, including cancer. Page 435

